# Comparative Evaluation of Hematopoietic Stem Cell Cryopreservation Using Liquid Nitrogen Versus -80°C Storage

**DOI:** 10.7759/cureus.112643

**Published:** 2026-07-14

**Authors:** Zakaria El Kodmiri, Maryame Ahnach, Sarah Kandoussi, Bouchra Ghazi, Jalila El Bakkouri

**Affiliations:** 1 Immunopathology, Immunotherapy, and Immunomonitoring Laboratory, Faculty of Medicine, Université Mohammed VI des Sciences et de la Santé (UM6SS), Casablanca, MAR

**Keywords:** -80 °c storage, cd34+, cell viability, cryopreservation, hematopoietic stem cells, liquid nitrogen

## Abstract

Hematopoietic stem cell (HSC) cryopreservation is a critical component of cellular therapy, directly influencing transplant outcomes. Although mechanical freezing at -80°C is widely used in resource-limited settings, liquid nitrogen (LN₂) cryopreservation with controlled-rate freezing remains the gold standard for long-term storage. However, comparative data on the efficacy and operational feasibility of these methods remain scarce. This study aimed to compare the efficacy, cellular integrity, and post-thaw CD34⁺ cell recovery of HSC grafts preserved using LN₂ cryopreservation versus -80°C mechanical freezing. Additionally, we sought to validate a controlled-rate freezing protocol and evaluate its clinical applicability within a cell therapy unit.

We conducted four experimental trials: (i) validation of a controlled-rate freezing protocol, (ii) assessment of post-thaw recovery in low-cell-density samples, and (iii-iv) a direct comparison between LN₂ and -80°C cryopreservation using paired aliquots. Post-thaw CD34⁺ cell counts were analyzed by flow cytometry using a BD FACSCanto™ II flow cytometer (Becton, Dickinson and Company, Franklin Lakes, NJ, USA), and freezing curves were recorded using a Consarctic® controlled-rate freezer (Consarctic GmbH, Schöllkrippen, Bavaria, Germany) to assess temperature descent consistency.

Both cryopreservation methods demonstrated measurable short-term post-thaw CD34⁺ cell recovery. In the comparative experiment, the absolute post-thaw CD34⁺ cell loss was similar (approximately 2 × 10⁶ cells/kg) between the two preservation methods. However, because baseline CD34⁺ cell contents differed between grafts, percentage recovery also differed, and recovery should therefore be interpreted cautiously. Liquid nitrogen cryopreservation provided superior thermal control, as demonstrated by strict adherence to the programmed freezing curve, and eliminated reliance on hydroxyethyl starch (HES) (Voluven®; Fresenius Kabi AG, Bad Homburg, Germany) as a cryoprotective component. No cryobag leakage or contamination was observed during the study. Given the exploratory pilot design and limited sample size, these findings should be considered preliminary and require confirmation in larger studies incorporating direct viability assays and functional assessments.

While -80°C cryopreservation remains a viable short-term option, LN₂ cryopreservation offers distinct advantages in thermal stability, cryoprotectant flexibility, and long-term cell viability. Our validated LN₂ protocol meets clinical standards for safety, sustainability, and quality, solidifying its role as the preferred method for high-quality HSC biobanking and transplantation. Further studies incorporating functional assays and extended follow-up are needed to assess long-term engraftment potential.

## Introduction

Cryopreservation of hematopoietic stem cells (HSCs) plays a central role in modern transplantation medicine. HSCs are multipotent progenitor cells capable of self-renewal and differentiation into all blood cell lineages and are used to restore hematopoiesis in patients undergoing autologous or allogeneic stem cell transplantation for hematological malignancies and other disorders. Clinically, CD34⁺ cells are widely used as a surrogate marker of transplantable HSCs and are routinely quantified to assess graft adequacy before transplantation. These cells must retain their biological integrity throughout collection, cryopreservation, storage, and thawing to ensure successful engraftment [1].

During cryopreservation, cells are exposed to osmotic stress, intracellular ice crystal formation, and membrane damage, all of which may impair post-thaw cellular recovery and function. Controlled-rate freezing minimizes these deleterious effects by gradually lowering the temperature before transfer to long-term storage, thereby reducing intracellular ice formation and improving cryopreservation quality. While mechanical freezing at -80°C has traditionally been used because of its lower cost and simpler infrastructure, particularly in resource-limited settings, liquid nitrogen (LN₂) (-196°C) cryopreservation with controlled-rate freezing is considered the clinical standard for long-term preservation owing to its superior thermal stability and ability to maintain cellular integrity during prolonged storage [2].

Reliable cryopreservation is essential because patients undergoing HSC transplantation depend on the recovery of sufficient functional CD34⁺ cells after thawing to achieve successful engraftment and hematopoietic reconstitution [3]. Inadequate cryopreservation may compromise graft quality, delay hematopoietic recovery, and adversely affect transplant outcomes. Therefore, selecting an appropriate cryopreservation strategy remains an important aspect of clinical stem cell processing [4].

Although recent advances in cryobiology have improved freezing protocols, direct comparative studies between liquid nitrogen and -80°C storage for HSCs remain limited [5]. Existing evidence primarily focuses on long-term storage outcomes or individual preservation strategies, while comparative evaluations performed under standardized experimental conditions remain scarce. Moreover, practical considerations such as equipment requirements, cryoprotectant availability, protocol reproducibility, and implementation within cell therapy laboratories have received comparatively little attention.

Therefore, this exploratory pilot study aimed to evaluate the feasibility, efficacy, and reliability of implementing controlled-rate liquid nitrogen cryopreservation within our cell therapy unit. The study consisted of three sequential experimental phases comprising four experimental trials designed to validate the controlled-rate freezing protocol, evaluate cryobag integrity and post-thaw CD34⁺ cell recovery, and descriptively compare liquid nitrogen and -80°C cryopreservation under standardized experimental conditions.

This article was previously posted as a preprint on the Research Square preprint server on September 19, 2025, and has not been published in a peer-reviewed journal.

## Technical report

Methods and materials

The cryopreservation procedures for liquid nitrogen and -80°C storage required specific equipment and consumables to ensure standardized processing of HSC grafts. Although the overall workflow was similar for both methods, differences were observed in the cryoprotective agents and freezing materials. Tables 1-2 summarize the equipment and consumables required for each protocol and highlight the principal methodological differences, particularly the cryoprotective formulations and freezing materials used for liquid nitrogen and -80°C cryopreservation.

**Table 1:** Equipment and consumables for cryopreservation with liquid nitrogen

Equipment	Consumables
Sterile connection device, +4°C refrigerator, Class 100 vertical laminar flow hood (PSM), Precision scale, Tube sealer, Forceps, Refrigerated bag centrifuge, Plasma press, Scissors, Stainless steel strainer, Stainless steel trays, Cooling blocks, Cardboard cassette, Sterile drape, Manual tube stripper	18G needle, 20% albumin stored at +4°C, 70% alcohol, Pediatric blood culture flask, Povidone-iodine, Sterile compresses, Cryotubes, DMSO (10 mL), Labels, Surgical gloves, Sterile gloves, Blades for sterile connection, 100ml freezing bags, 600 mL transfer bags, Sampling equipment, Syringes (60 mL, 10 mL, 5 mL, 1 mL) with needles, 0.9% NaCl

**Table 2:** Equipment and consumables for cryopreservation at -80°C

Equipment	Consumables
Sterile connection device, +4°C refrigerator, Class 100 vertical laminar flow hood (PSM), Precision scale, Tube sealer, Forceps, Refrigerated bag centrifuge, Plasma press, Scissors, Stainless steel strainer, Stainless steel trays, Cooling blocks, Cardboard cassette, Sterile drape, Manual tube stripper	18G needle, 20% albumin stored at +4°C, 70% alcohol, Pediatric aerobic blood culture flask, Povidone-iodine, Sterile compresses, Cryotubes, DMSO (10 mL, Labels, Surgical gloves, Sterile gloves, Blades for sterile connection, Freezing bags (60 mL or 100 mL), 600 mL transfer bags, Sampling equipment, Syringes (60 mL, 10 mL, 5 mL, 1 mL) with needles, Voluven (hydroxyethyl starch)

Because this study was designed as an exploratory pilot investigation based on single experimental observations for each trial, no formal statistical analyses or measures of variability were performed. Accordingly, the results are presented descriptively, and no statistical comparisons between cryopreservation methods were undertaken. The study consisted of three sequential experimental phases encompassing four experimental trials. The HSC grafts evaluated were autologous peripheral blood stem cell (PBSC) collections obtained by leukapheresis following mobilization according to institutional practice. Trial 1 validated the controlled-rate freezing protocol and freezing equipment. Trial 2 assessed post-thaw CD34⁺ cell recovery using a low-CD34⁺-content graft. Trials 3 and 4 compared liquid nitrogen and -80°C cryopreservation using paired graft aliquots processed under identical conditions. Baseline CD34⁺ cell content for each trial is reported in the Results section.

Trial 1: Freezing Protocol Validation and Temperature Gradient Evaluation

This trial aimed to validate the controlled-rate freezing protocol using the Consarctic® freezer (Consarctic GmbH, Schöllkrippen, Bavaria, Germany) and to assess cryobag integrity during liquid nitrogen storage. A HSC bag was processed through a standardized freezing cycle, beginning with gradual cooling at -1°C/min to -4°C, followed by manual seeding and subsequent cooling phases (-1°C/min to -40°C, then -5°C/min to -125°C) before transfer to vapor-phase liquid nitrogen storage (-196°C) in tank PS151. After 63 days of cryopreservation, the bag was thawed and evaluated with no observed bag leakage and full equipment functionality. 

Trial 2: Low-Concentration HSC Recovery Assessment

The freezing process was conducted using liquid nitrogen at -196°C with 10% dimethyl sulfoxide (DMSO) (Merck KGaA, Darmstadt, Germany) as the cryoprotectant, following the validated protocol from the initial trial to assess the freezing procedure, verify the effectiveness of the gradual freezing cycle, and test cell recovery after thawing a low-cell-density bag. Post-thaw, a sample was analyzed by flow cytometry using a BD FACSCanto™ II flow cytometer (Becton, Dickinson and Company, Franklin Lakes, NJ, USA) to determine post-thaw CD34⁺ cell recovery by quantifying CD34⁺ cells.

Trials 3 and 4: Comparison of Preservation Methods

This study aimed to compare the effects of liquid nitrogen and -80°C cryopreservation on post-thaw CD34⁺ cell recovery in HSC grafts. An autologous HSC graft was divided into two equal-volume aliquots. Aliquot A was cryopreserved using the validated controlled-rate liquid nitrogen protocol, whereas Aliquot B was cryopreserved at -80°C according to the standard institutional procedure. Following thawing, each aliquot was analyzed independently by flow cytometry using a BD FACSCanto™ II flow cytometer to quantify CD34⁺ cell counts. Post-thaw evaluation consisted of CD34⁺ cell enumeration only; no direct viability assay (7-AAD or propidium iodide staining) was performed. Therefore, the study outcomes are reported as post-thaw CD34⁺ cell recovery rather than cell viability. This paired comparison design was intended to minimize donor-related variability and enable a descriptive comparison of post-thaw CD34⁺ cell recovery between the two cryopreservation methods.

Ethics Statement

This study was conducted in accordance with the principles of the Declaration of Helsinki and approved by the Ethics Committee of Université Mohammed VI des Sciences et de la Santé (UM6SS) (CE/UM6SS/36/26), Casablanca, Morocco. Ethical approval was granted on April 10, 2026. Given the retrospective nature of the study and the use of anonymized data, the requirement for informed consent was waived by the Ethics Committee.

Results

**Figure 1:**
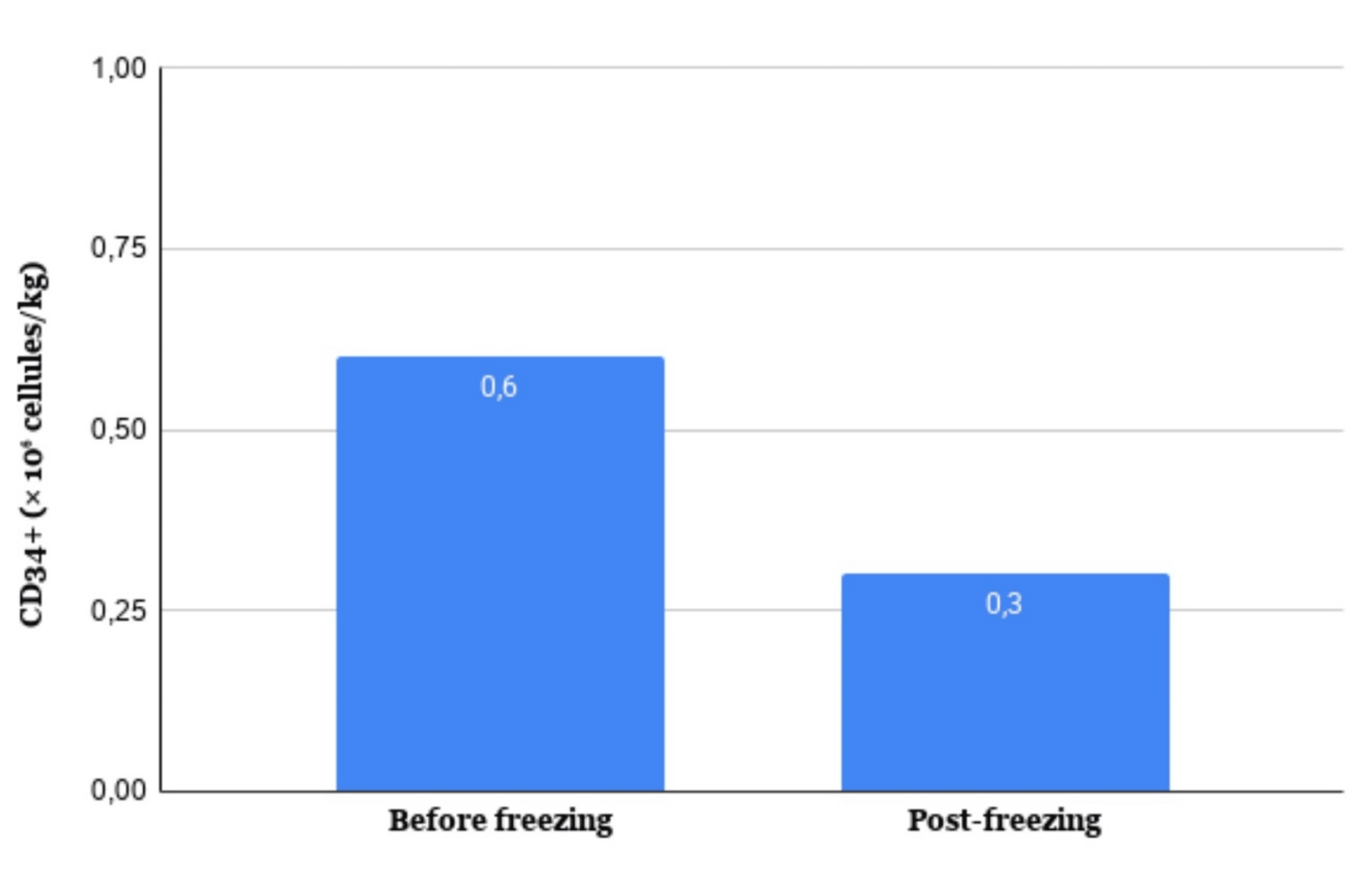
Comparison of pre- and post-thaw CD34⁺ cell content in the low-CD34⁺ graft (×10⁶ cells/kg), Trial 2 An HSC graft with an initial CD34⁺ cell content of 0.6 × 10⁶ cells/kg demonstrated a post-thaw CD34⁺ cell content of 0.3 × 10⁶ cells/kg following liquid nitrogen cryopreservation. HSC: hematopoietic stem cell

The validated controlled-rate freezing protocol was first evaluated using a low-cell-concentration HSC graft. CD34⁺ cell counts were determined before cryopreservation and immediately after thawing to assess cellular recovery. A decrease in CD34⁺ cell content was observed following cryopreservation, while detectable CD34⁺ cells remained after thawing. The quantitative comparison of pre- and post-thaw CD34⁺ cell concentrations is presented in Figure 1.

**Figure 2:**
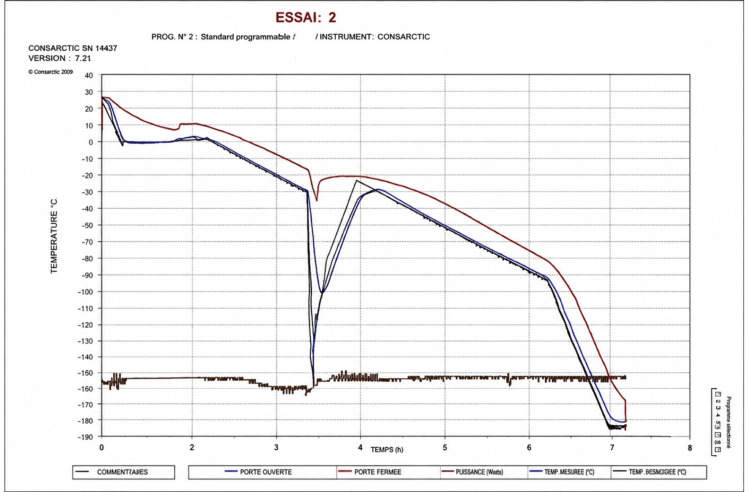
Temperature descent curve recorded during the freezing of the HSC bag - Trial 2 The temperature profile demonstrates reproducible adherence to the programmed controlled-rate freezing protocol using the Consarctic® freezer, from room temperature to -125°C before transfer to liquid nitrogen storage, supporting standardized cryopreservation conditions. HSC: hematopoietic stem cell

Percentage CD34+ recovery was calculated as the ratio of post-thaw to pre-freezing CD34+ cell counts and is reported descriptively for each experimental trial. To verify the performance of the freezing protocol, the temperature profile was continuously monitored throughout the controlled-rate freezing process. The recorded freezing curve demonstrates the programmed temperature descent from room temperature to the target freezing temperature before transfer to liquid nitrogen storage. The complete temperature profile obtained during Trial 2 is shown in Figure 2.

**Figure 3:**
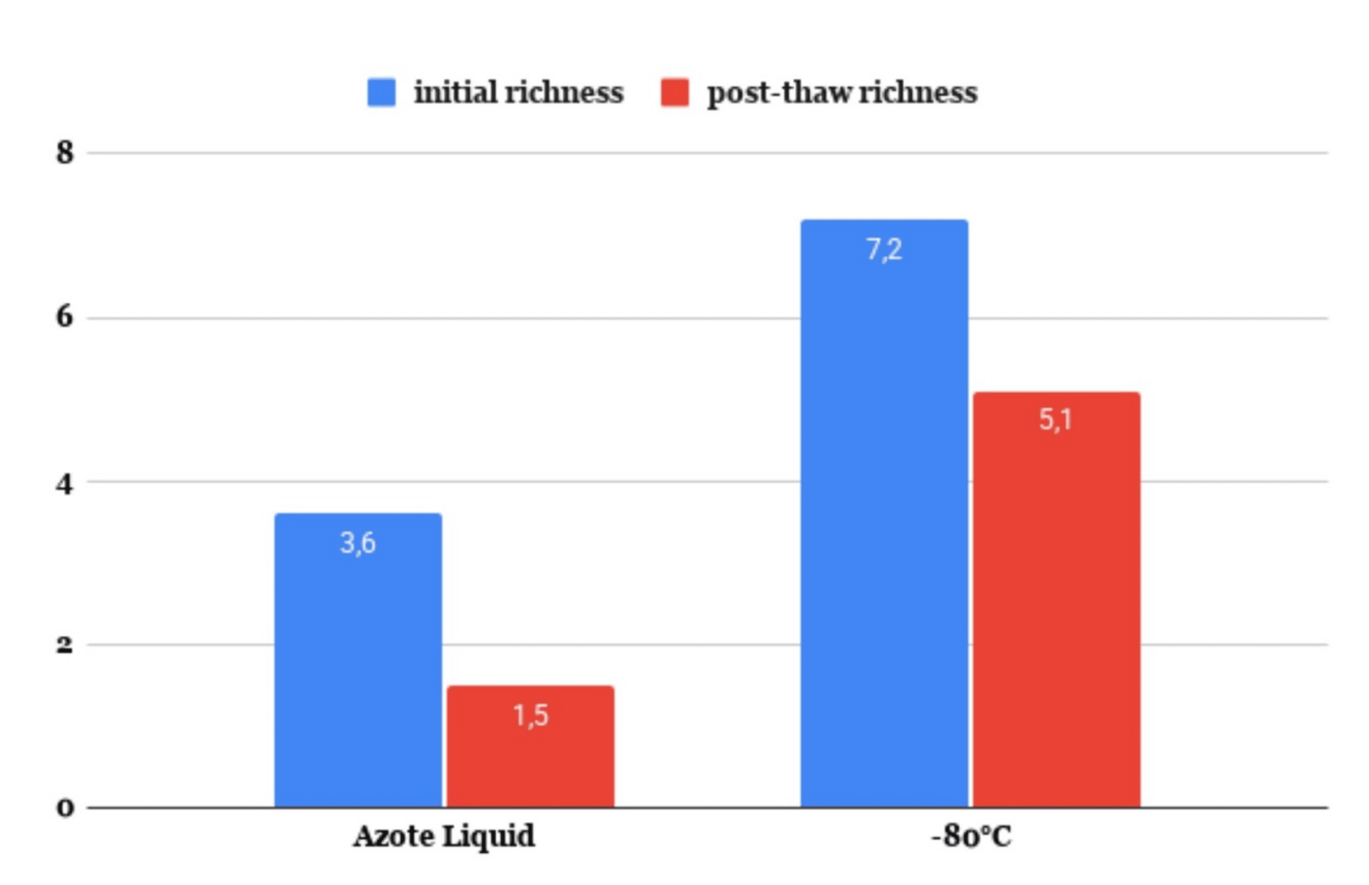
Comparison of post-thaw CD34⁺ cell recovery after 13 days of liquid nitrogen and -80°C cryopreservation In Trial 3, liquid nitrogen cryopreservation was performed on a graft with an initial CD34⁺ cell content of 3.6 × 10⁶ cells/kg, resulting in a post-thaw loss of 2.1 × 10⁶ cells/kg. In Trial 4, -80°C cryopreservation was performed on a graft with an initial CD34⁺ cell content of 7.2 × 10⁶ cells/kg, resulting in a post-thaw loss of 2.0 × 10⁶ cells/kg. Because baseline CD34⁺ cell contents differed between the paired aliquots, the findings are presented descriptively and should be interpreted with caution.

The performance of liquid nitrogen and conventional -80°C cryopreservation was subsequently evaluated using paired HSC aliquots processed under identical experimental conditions. Following thawing, CD34⁺ cell counts were measured to compare post-thaw cellular recovery between the two preservation methods. The corresponding quantitative results obtained for both cryopreservation approaches are presented in Figure 3.

**Figure 4:**
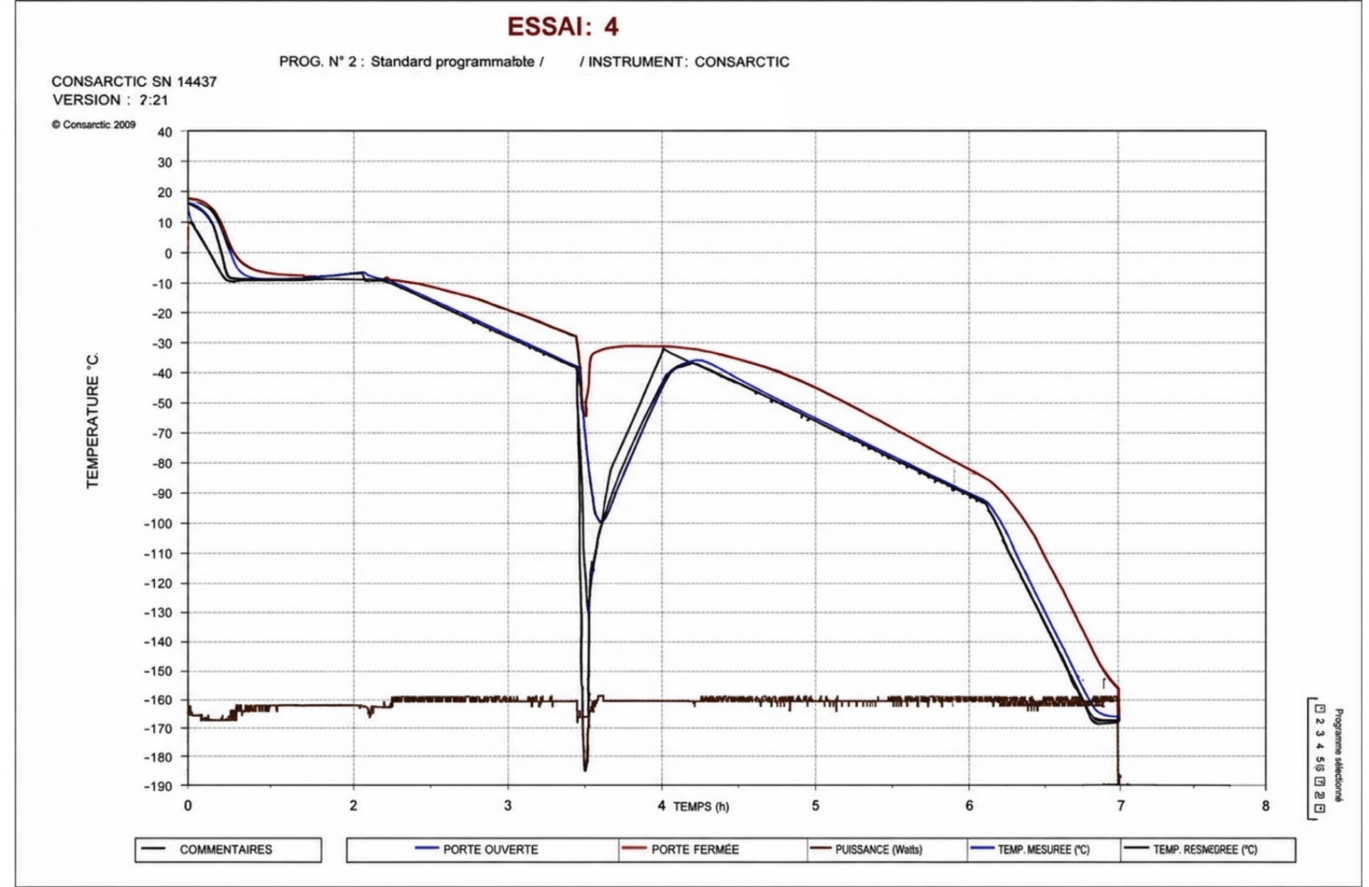
Temperature profile of HSC cryopreservation (liquid nitrogen method) - Trial 3 The curve shows a gradual and controlled temperature descent to approximately -120°C, in accordance with the protocol established using the Consarctic® freezer, before transfer into liquid nitrogen for cryostorage. This curve confirms the proper execution of the controlled-rate freezing process, which is essential for preserving graft integrity and optimizing post-thaw CD34⁺ cell recovery. The graft stored at -80°C was not subjected to continuous temperature monitoring but was cryopreserved according to the standard institutional protocol. HSC: hematopoietic stem cell

To assess the reproducibility of the controlled-rate freezing procedure during the comparative experiment, temperature monitoring was performed throughout the liquid nitrogen cryopreservation process. The recorded freezing profile illustrates the programmed cooling sequence achieved before transfer of the graft into liquid nitrogen storage. The temperature curve corresponding to this experiment is presented in Figure 4.

## Discussion

This work should be considered an exploratory pilot study designed to validate the implementation of a controlled-rate cryopreservation protocol and to generate preliminary comparative data. This study presents a comparative assessment of two cryopreservation methods: mechanical freezing at -80°C and storage in liquid nitrogen, for the preservation of HSCs. Our findings indicate that both techniques preserved measurable short-term post-thaw CD34⁺ cell recovery. However, differences were observed in thermal regulation, cryoprotectant requirements, and potential applicability for long-term cryostorage. As no direct viability assay was performed, the present findings are limited to post-thaw CD34⁺ cell recovery and should not be interpreted as direct measurements of cell viability or long-term functional preservation.

Short-term efficacy of mechanical freezing at -80°C

Analysis of post-thaw outcomes from Trials 3 and 4 showed similar absolute CD34⁺ cell loss following liquid nitrogen and -80°C cryopreservation. However, because baseline CD34⁺ cell contents differed between the two graft aliquots and no formal statistical analysis was performed, these findings should be interpreted descriptively. The results suggest that both cryopreservation methods preserved measurable post-thaw CD34⁺ cell recovery in this exploratory pilot study. These findings are consistent with those of Campos-Carli et al. (2024), who reported stable CD34⁺ cell recovery and successful transplantation outcomes following long-term -80°C cryopreservation when liquid nitrogen storage was not feasible [6].

Despite these findings, mechanical freezing exhibits notable limitations. Unlike controlled-rate freezing, it lacks precise thermal regulation, increasing the risk of intracellular ice crystal formation and subsequent cellular damage [7]. Additionally, the clinical scalability of -80°C protocols is constrained by the limited global availability of Voluven® (hydroxyethyl starch, HES) (Fresenius Kabi AG, Bad Homburg, Germany), a key extracellular cryoprotectant in many clinical-grade formulations. However, HES has been associated with allergic reactions [8]. While adjunctive agents such as Albumin have demonstrated improved thermal stability and reduced recrystallization in experimental settings, their use remains non-standardized for clinical HSC cryopreservation and has primarily been validated in pluripotent stem cell models to improve regenerative therapies [9,10]. Thus, while mechanical freezing may serve as a provisional alternative in resource-limited settings, its long-term reliability and reproducibility remain uncertain. Further investigations incorporating functional assays such as colony-forming unit (CFU) analysis and in vivo hematopoietic reconstitution models are necessary to comprehensively evaluate the regenerative potential of -80°C-preserved HSCs [11].

Liquid nitrogen cryopreservation: the gold standard

In contrast, LN₂ cryopreservation, particularly when preceded by controlled-rate freezing, remains the gold standard for long-term HSC storage. The gradual temperature reduction minimizes intracellular ice formation, preserves membrane integrity, and mitigates cellular stress. As demonstrated by the curve (Figure 2), the stability and consistency of the freezing cycle are confirmed. These are essential factors for ensuring post-thaw CD34⁺ recovery. Furthermore, as shown by the freezing curve in Figure 4, our data support established cryobiological principles, confirming that controlled-rate freezing followed by ultra-low-temperature (-196°C) storage is critical for maintaining HSC structural and functional integrity. Hornberger et al. (2019) emphasized that such conditions halt metabolic activity, preserving HSC clonogenicity and engraftment potential for over a decade [12]. Furthermore, their review underscores the importance of combining DMSO with extracellular cryoprotectants (HES) to reduce DMSO-associated cytotoxicity, an approach that was implemented in our study to enhance post-thaw viability while sustaining long-term stem cell function [12]. From a biosafety perspective, we addressed potential microbial contamination risks in LN₂ storage by utilizing sterile, single-use protective packaging, ensuring cryobag integrity throughout storage and handling.

Clinical and logistical considerations

When selecting cryopreservation methods for clinical and biobanking applications, infrastructure, reagent availability, and protocol standardization are critical factors [13]. Although -80°C systems offer logistical and cost advantages, their reliance on non-standardized cryoprotectants (e.g., Voluven®) and the absence of long-term clinical validation for HSCs restrict their widespread adoption [14]. Conversely, LN₂-based systems provide a well-established, standardized platform for long-term HSC preservation [14]. Their compatibility with controlled-rate freezing and their capacity for large-volume cryobanking reinforce their clinical superiority. Moreover, as demonstrated in this study, LN₂ protocols can be optimized to mitigate contamination risks, enhancing their suitability for both research and therapeutic applications [15].

Future directions

While our results suggest that both -80°C and LN₂ cryopreservation preserved measurable short-term post-thaw CD34⁺ cell recovery, functional assessments are essential to evaluate long-term regenerative capacity. Future research should prioritize functional characterization through clonogenic (CFU) assays and in vivo hematopoietic reconstitution studies, alongside comparative clinical analyses of engraftment efficiency in transplant recipients [16,17]. Additionally, economic and logistical evaluations of cryoprotectant utilization across preservation platforms should be conducted [18,19]. Further efforts should focus on optimizing -80°C protocols for clinical application, including the standardization of cryoprotectant formulations and validation of long-term storage efficacy.

Study limitations

This study has several limitations. First, functional assessments (clonogenic potential, engraftment capacity) were not performed despite evaluating post-thaw CD34 recovery. Second, the study included only a single experimental observation for each trial, precluding statistical analysis and limiting the generalizability of the findings. Third, the -80°C protocol's Voluven® dependence affects reproducibility. Additionally, post-thaw immunophenotypic changes were not assessed. Finally, longer-term storage effects, especially for -80°C preservation, require further study.

## Conclusions

This exploratory pilot study demonstrates the feasibility of implementing a controlled-rate liquid nitrogen cryopreservation protocol for HSC grafts within a cell therapy unit. Both liquid nitrogen and -80°C cryopreservation preserved measurable short-term post-thaw CD34⁺ cell recovery, while the controlled-rate liquid nitrogen protocol provided reproducible thermal control and maintained cryobag integrity throughout the cryopreservation process.

However, as only short-term post-thaw CD34⁺ cell recovery was evaluated, no direct viability assays, functional assays, or long-term clinical outcomes were assessed. Therefore, the present findings should not be interpreted as evidence of superior long-term cell viability, preserved stem cell function, or overall superiority of one cryopreservation method over the other. While liquid nitrogen cryopreservation remains the current clinical standard for long-term stem cell banking according to the existing literature, the findings of this exploratory pilot study should be considered preliminary. Larger prospective studies incorporating standardized viability assays, CFU assays, long-term storage evaluation, and clinical engraftment outcomes are required to confirm these observations and establish the comparative performance of the two cryopreservation strategies.

## References

[1] Berz D, McCormack EM, Winer ES, Colvin GA, Quesenberry PJ (2007). Cryopreservation of hematopoietic stem cells. Am J Hematol.

[2] Prisciandaro M, Santodirocco M (2024). Innovation in hematopoietic stem cell cryopreservation and cold chain management. Cell Gene Ther Insights.

[3] Bennett B, Hanotaux J, Pasala AR (2024). Impact of lower concentrations of dimethyl sulfoxide on cryopreservation of autologous hematopoietic stem cells: a systematic review and meta-analysis of controlled clinical studies. Cytotherapy.

[4] Mancias-Guerra C, Valdés-Burnes SL, Gonzalez-Llano O (2013). Another method for thawing hematopoietic stem cells and its impact in the recovery of the transplanted hematological patient. Biol Blood Marrow Transplant.

[5] Desoutter J, Ossart C, Lacassagne MN, Regnier A, Marolleau JP, Harrivel V (2019). Cryopreservation and thawing of hematopoietic stem cell CD34-induced apoptosis through caspase pathway activation: key role of granulocytes. Cytotherapy.

[6] Campos-Carli SM, Filho OAM, Avelar DMV (2024). Cryopreservation of hematopoietic stem cells at -80°C: how long is it safe?. Hematol Transfus Cell Ther.

[7] Gomes MRA, Ramos AS, Alves ACL (2024). Validation of the use of hydroxyethyl starch (Voluven 6%) to remove dimethyl sulfoxide from hematopoietic stem cells. Hematol Transfus Cell Ther.

[8] Lee JY, Hong SH (2020). Hematopoietic stem cells and their roles in tissue regeneration. Int J Stem Cells.

[9] Kuten Pella O, Hornyák I, Horváthy D, Fodor E, Nehrer S, Lacza Z (2022). Albumin as a biomaterial and therapeutic agent in regenerative medicine. Int J Mol Sci.

[10] Wang J, Ye D, Li S (2024). Generation of human induced pluripotent stem cells carrying albumin-sfGFP reporter. Stem Cell Res.

[11] Jahan S, Kaushal R, Pasha R, Pineault N (2021). Current and future perspectives for the cryopreservation of cord blood stem cells. Transfus Med Rev.

[12] Hornberger K, Yu G, McKenna D, Hubel A (2019). Cryopreservation of hematopoietic stem cells: emerging assays, cryoprotectant agents, and technology to improve outcomes. Transfus Med Hemother.

[13] Yuan X, Yuan F, Wang Y (2016). Efficient long-term cryopreservation of pluripotent stem cells at −80°C. Sci Rep.

[14] Gokarn A, Tembhare PR, Syed H (2023). Long-term cryopreservation of peripheral blood stem cell harvest using low concentration (4.35%) dimethyl sulfoxide with methyl cellulose and uncontrolled rate freezing at -80 °C: an effective option in resource-limited settings. Transplant Cell Ther.

[15] Lecchi L, Giovanelli S, Gagliardi B, Pezzali I, Ratti I, Marconi M (2016). An update on methods for cryopreservation and thawing of hemopoietic stem cells. Transfus Apher Sci.

[16] Fountain D, Ralston M, Higgins N (1997). Liquid nitrogen freezers: a potential source of microbial contamination of hematopoietic stem cell components. Transfusion.

[17] Sarma NJ, Takeda A, Yaseen NR (2010). Colony forming cell (CFC) assay for human hematopoietic cells. J Vis Exp.

[18] Maqbool S, Nadeem M, Shahroz A (2022). Engraftment syndrome following Hematopoietic stem cell transplantation: a systematic approach toward diagnosis and management. Med Oncol.

[19] Turney TL, Reese-Koç J, de Lima M, Otegbeye F (2019). Optimizing cryopreservation of hematopoietic stem cells collected for autologous stem cell transplantation in patients with multiple myeloma. Cytotherapy.

